# Early and Long-Term Follow-Up for Chronic Type B and Type Non-A Non-B Aortic Dissection Using the Frozen Elephant Trunk Technique

**DOI:** 10.3389/fcvm.2021.714638

**Published:** 2021-09-14

**Authors:** Congcong Luo, Ruidong Qi, Yongliang Zhong, Suwei Chen, Hao Liu, Rutao Guo, Yipeng Ge, Lizhong Sun, Junming Zhu

**Affiliations:** Department of Cardiovascular Surgery, Beijing Aortic Disease Center, Beijing Anzhen Hospital, Capital Medical University, Beijing, China

**Keywords:** non-A non-B aortic dissection, chronic type B aortic dissection, frozen elephant trunk technique, total arch replacement, long term outcomes

## Abstract

**Background:** This study aimed to evaluate the early and long-term outcomes of a single center using a frozen elephant trunk (FET) procedure for chronic type B or non-A non-B aortic dissection.

**Methods:** From February 2009 to December 2019, 79 patients diagnosed with chronic type B or non-A non-B aortic dissection who underwent the FET procedure were included in the present study. We analyzed operation mortality and early and long-term outcomes, including complications, survival and interventions.

**Results:** The operation mortality rate was 5.1% (4/79). Spinal cord injury occurred in 3.8% (3/79), stroke in 2.5% (2/79), and acute renal failure in 5.1% (4/79). The median follow-up time was 53 months. The overall survival rates were 96.2, 92.3, 88.0, 79.8, and 76.2% at 1/2, 1, 3, 5 and 7 years, respectively. Moreover, 79.3% of patients did not require distal aortic reintervention at 7 years. The overall survival in the subacute group was superior to that in the chronic group (*P* = 0.047).

**Conclusion:** The FET technique is a safe and feasible approach for treating chronic type B and non-A non-B aortic dissection in patients who have contraindications for primary endovascular aortic repair. The technique combines the advantages of both open surgical repair and endovascular intervention, providing comparable early and long-term follow-up outcomes and freedom from reintervention.

## Introduction

According to current expert consensus, patients with uncomplicated type B aortic dissection (AD) are suggested for medical treatment and periodic clinical and imaging surveillance. Surgical repair and interventional treatment are considered options for complicated type B or non-A non-B AD (1–3). Recently, thoracic endovascular aortic repair (TEVAR) has been recommended as the first-line treatment for complicated type B or non-A non-B AD because of its lower morbidity and mortality (4). However, TEVAR may not be feasible for patients who have an unfavorable aortic anatomy, a lack of a sufficient proximal landing zone, a high risk of retrograde type A aortic dissection (RTAAD) and/or concomitant with a proximal aortic lesion.

The frozen elephant trunk (FET) procedure may be an alternative treatment for these kinds of patients, according to the recent recommendations published by the European Society for Vascular Surgery and the European Association for Cardio-Thoracic Surgery (5, 6). The FET procedure combines the advantages of open surgery and endovascular treatment, by which total arch replacement (TAR) and descending aortic dissection repair (7, 8) can be performed simultaneously. The FET procedure can eliminate the risk of type Ia endoleak and RTAAD, benefit the thrombosis of false lumen, enable positive aortic remodeling and improve prognosis (9). However, there have been few reports on the outcomes of chronic type B or non-A non-B AD using the FET procedure, and the long-term outcomes, in particular, have not been reported (10).

Therefore, we retrospectively reviewed the experience in our center on the treatment of complicated type B or non-A non-B aortic dissection using the FET procedure according to the most recent published recommendations (5, 6). This study was conducted in accordance with the rules and checklist of the STROBE statement.

## Patients and Methods

This study was performed in accordance with the Declaration of Helsinki (2013). The Ethics Committees of Beijing Anzhen Hospital, Capital Medical University, approved this retrospective study (2020100X).

### Patients

From February 2009 to December 2019, 79 consecutive patients diagnosed with chronic type B or non-A non-B aortic dissection underwent open surgical repair using TAR combined with FET under hypothermic cardiopulmonary bypass (CPB) and antegrade selective cerebral perfusion (ASCP) via median sternotomy in Beijing Anzhen Hospital. Computed tomographic angiography (CTA) and echocardiography were performed to evaluate and confirm the diagnosis preoperatively.

According to the definition of the STROAGE guidelines, there were 60 subacute dissections (75.9%, 14–90 days from the appearance of symptoms) and 19 chronic dissections (24.1%, >90 days) (11). The mean age was 45.0 ± 13.1 years, and 54 patients were male (68.4%). Hypertension was the most frequent risk factor in this cohort, which was observed in 49 patients (62.0%). Marfan syndrome was noted in 14 (17.7%), along with coronary artery disease in 5 (6.3%), chronic heart failure in 1 (1.3%), respiratory disease and prior cerebrovascular accident in 2 each (2.5%) and chronic kidney disease in 1 (1.3%). Sixty-two patients (78.5%) had ascending/root aortic aneurysms, aortic arch aneurysms in 9 cases (11.5%), severe aortic valve regurgitation in 23 cases (29.5%) and severe mitral valve regurgitation in 3 cases (3.8%). Four patients had undergone a previous aortic procedure, including the Bentall procedure in 3 (3.8%), mitral valvuloplasty in 1 (1.3%), ascending aortic replacement in 1 (1.3%) and abdominal aortic replacement in 1 (1.3%). More detailed parameters are presented in Table 1.

**Table 1:** Preoperative parameters and comorbidities.

**Variables**	**Total (** ***n*** **= 79)**	**Subacute (** ***n*** **= 60)**	**Chronic (** ***n*** **= 19)**	***P*** **-value**
Time from onset to surgery (d)	33 (21–81)	26.5 (20–37)	270 (132–790)	
Type B AD	32 (40.5)	21 (35.0)	11 (57.9)	0.076
Non-A non-B AD	47 (59.5)	39 (65.0)	8 (42.1)	0.076
Male	54 (68.4)	41 (68.3)	11 (68.4)	0.994
Age (y)	45.0 ± 13.1	45.8 ± 12.6	42.1 ± 14.3	0.275
Smoking	30 (38.0)	25 (41.7)	5 (26.3)	0.230
**Comorbidities**				
Hypertension	49 (62.0)	36 (60.0)	13 (68.4)	0.510
Marfan syndrome	14 (17.7)	8 (13.3)	6 (31.6)	0.141
Coronary artery disease	5 (6.3)	4 (6.7)	1 (5.3)	0.827
Chronic heart failure	1 (1.3)	1 (1.7)	0	0.759
Prior cerebrovascular accident	2 (2.5)	2 (3.3)	0	0.574
COPD	2 (2.5)	1 (1.7)	1 (5.3)	0.426
Chronic kidney disease	1 (1.3)	0	1 (5.3)	0.241
Gout	1 (1.3)	0	1 (5.3)	0.241
**Proximal lesion**				
Aortic arch aneurysm	9 (11.5)	4 (6.8)	5 (26.3)	0.163
Aortic root/ascending aneurysm	62 (78.5)	45 (75.0)	17 (89.5)	0.219
Type A IMH	6 (7.6)	5 (8.3)	1 (5.3)	0.648
Severe AR	23 (29.5)	17 (28.8)	6 (31.6)	0.818
Severe MR	3 (3.8)	3 (5.1)	0	0.427
Crawford type I aneurysm	2 (2.6)	2 (3.4)	0	0.570

*IQR, interquartile range; SD, standard deviation; IMH, intramural hematoma; AR, aortic regurgitation; MR, mitral regurgitation; COPD, chronic obstructive pulmonary disease*.

### Surgical Technique

The detailed surgical technique of TAR combined with FET was described previously (12, 13). Island technique arch reconstruction was performed when there was no involvement of the brachiocephalic trunk and the left common carotid artery. Detailed procedures were described previously (14, 15).

The length of the stented graft (MicroPort Medical Co Ltd, Shanghai, China) was 10 cm, the diameter was 24–30 mm, and the graft had 3 and 1-cm stent-free vascular grafts for sutures in the proximal and distal edges, respectively. The size of the stented graft was determined according to the diameter of the proximal descending aorta of healthy individuals matched for age, sex, and height. The diameter of the stented graft was larger than the true lumen but slightly smaller than the entire chosen aorta.

Briefly, after a median sternotomy, the brachiocephalic vessels and the transverse arch were dissected and exposed. The left sternocleidomastoid muscle and other cervical muscle groups were partially transected if necessary. The left subclavian artery (LSCA) and the left common carotid artery (LCCA) were carefully separated and exposed, and care was taken to avoid injury to the thoracic duct; then, the thoracic duct was ligated if necessary.

Right axillary artery cannulation was used for CPB and ASCP. Cooling was initiated when CPB was established. Cold hyperkalemic cardioplegic solution was used for cardiac arrest. Then, valvular repair or replacement and proximal aortic operations were performed during the cooling phase. When the nasopharyngeal temperature was under 25°C, circulatory arrest was performed. Unilateral ASCP was initiated with a flow rate of 5–10 mL·kg^−1^·min^−1^ by the right axillary artery. Unilateral ASCP was considered to be adequate for cerebral circulation in the left hemisphere when the left radial artery pressure was 20 mmHg and there was recurrent bleeding through the LCCA; otherwise, bilateral ASCP was performed.

**Figure 1:**
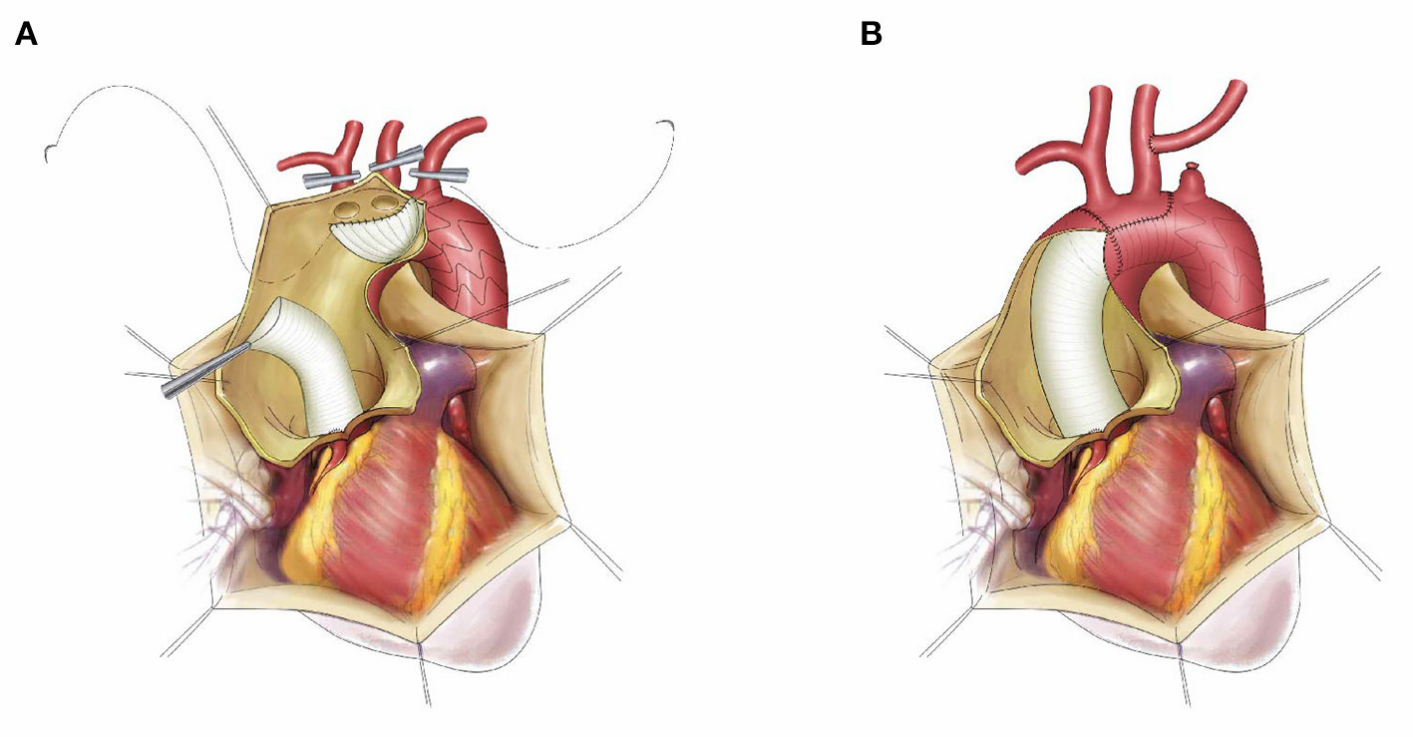
The schematic diagram of frozen elephant trunk with “island technique” and left subclavian aorta to left common carotid artery transposition. The stented graft was inserted into the true lumen of descending aorta, the stent-free vascular graft was trimmed to a semi-oval shape containing the innominate artery and left common carotid artery, then the inner suture was performed counterclockwise while the outer suture was performed clockwise **(A)**; The final result after suture, left subclavian aorta to left common carotid artery transposition **(B)**.

The anterior wall of the aortic arch was incised up to the origin of the LCCA, and the incision was performed ~0.5 cm distal to the origin of the IA and the LCCA. No dissection of the innominate artery (IA) or the LCCA was confirmed intraoperatively. The stented graft was inserted into the true lumen of the descending aorta and deployed. The stent-free vascular graft was pulled and trimmed to a semioval shape to match the aortic arch wall containing the IA and the LCCA. A running suture was performed within the stent-free vascular graft between the LSCA and the LCCA using 4-0 Prolene, and the inner suture was performed counterclockwise while the outer suture was performed clockwise. Thus, the residual aortic wall and the stent-free vascular graft formed a circular opening. Finally, the distal end of the ascending aortic graft was anastomosed to the circular opening using open distal anastomosis. After anastomosis, the CPB gradually resumed to normal flow, and rewarming was started. The left subclavian artery was transected 0.5–1.0 cm distal to the origin, and the proximal segment was sutured using 5-0 Prolene. The LSCA was anastomosed to the LCCA in an end-to-side fashion (Figure 1).

### Study Endpoints and Follow-Up

The primary study endpoints were operation death and late death. Overall in-hospital mortality was defined as death within 30 days after surgery and hospital death. Late mortality was defined as all-cause death beyond 30 days after surgery during follow-up. Secondary endpoints included distal aortic reoperations and complications. Distal aortic reoperation referred to any reinterventions, including open surgeries or endovascular interventions on the distal aorta. Complications included stroke, endoleak, limb ischemia and spinal cord injury.

**Figure 2:**
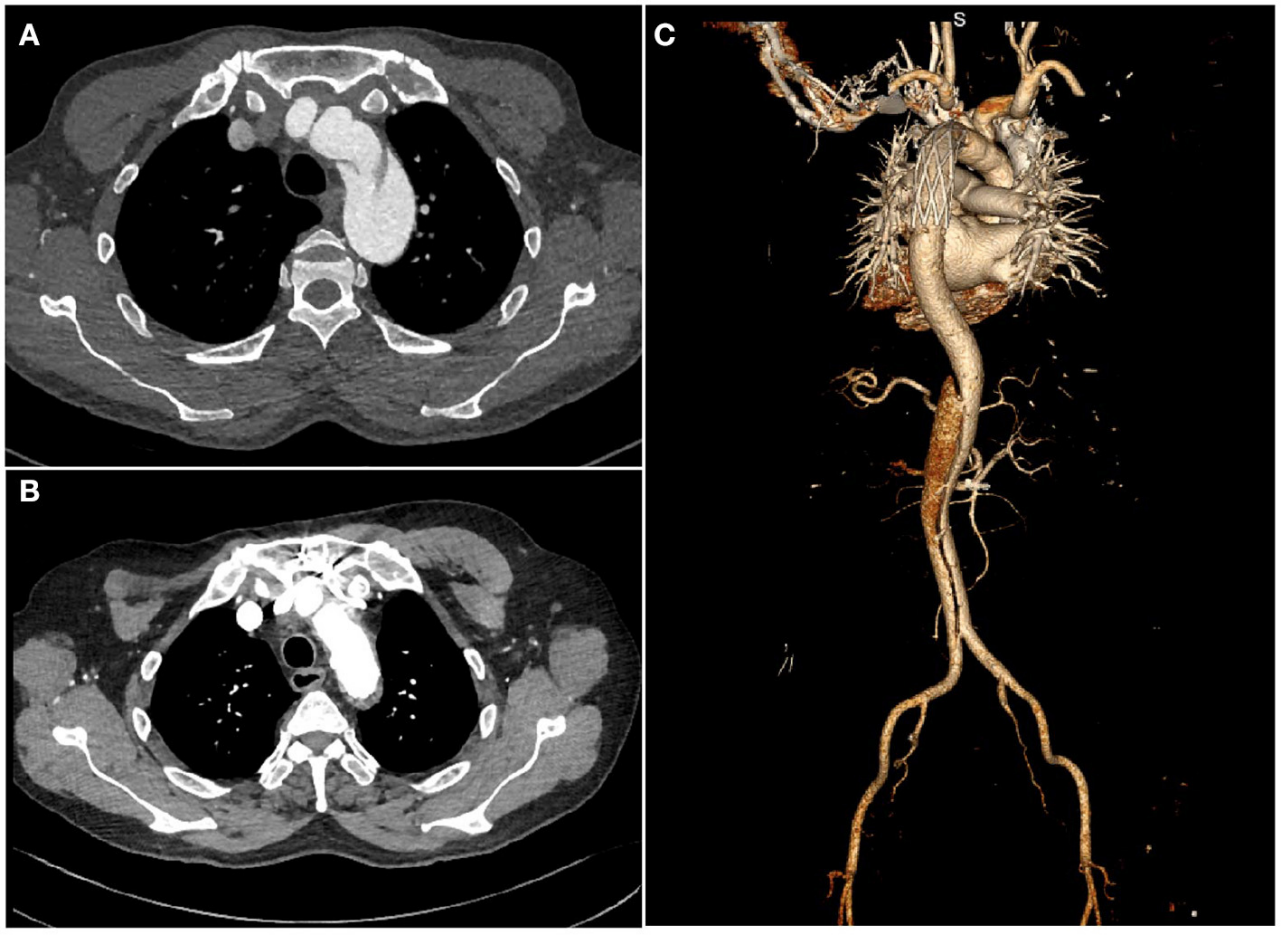
Computed tomography images of a patient with chronic type B dissection with aortic arch involvement **(A)** preoperatively **(B,C)**, postoperatively.

All discharged patients were followed up regularly through clinic visits, phone calls, emails or letters. All survivors were recommended to undergo periodic CTA scans of the entire aorta (Figure 2).

### Statistical Analysis

All analyses in the study were performed using SPSS software version 25.0 (IBM Corporation, Chicago, USA) and GraphPad Prism for Windows 8.0 (La Jolla, CA, USA.). Continuous variables are summarized as the mean ± standard deviation (SD) or median with interquartile range (IQR) according to their normality and were compared using Student's *t*-test or the Mann-Whitney U test. Categorical variables are expressed as numbers (percentage) and were compared using the Pearson χ^2^ test or Fisher's exact test. Survival analysis and freedom from distal aortic reoperation were estimated using the Kaplan-Meier method. Competing risk analysis was performed based on the methods described by Blackstone et al. (16). A 2-sided significance level with a *P* < 0.05 was considered statistically significant.

## Results

### Surgical Data

All patients underwent elective surgery and completed it successfully. Most patients (59.5%) underwent “island technique” aortic arch branch anastomosis (Table 2). Approximately half of the patients (54.4%) required the Bentall procedure, 23 patients (29.1%) underwent ascending aortic replacement, and 3 patients (3.8%) underwent the David procedure. Other concomitant procedures included mitral valve repair in 3 patients (3.8%) and coronary artery bypass grafting in 5 patients (6.3%). Extra anatomic bypass occurred in 4 cases (5.1%), including ascending aorta-femoral aorta bypass in 2 patients (2.8%), axillary artery-axillary artery bypass in 1 patient and ascending aorta-axillary artery bypass in 1 patient (1.4%).

**Table 2:** Intraoperative data.

**Variables**	**Total (** ***n*** **= 79)**	**Subacute (*n =* 60)**	**Chronic (** ***n*** **= 19)**	***P*** **-value**
Aortic cross-clamp time (min)	89 (75–119)	89 (72–115)	100 (83–124)	0.233
Rectal temperature (°C)	25.3 ± 2.0	25.2 ± 2.0	25.7 ± 1.7	0.376
Nasopharyngeal temperature (°C)	23.7 (22.3–24.4)	23.7 (22.2–24.4)	23.9 (22.3–24.4)	0.528
Circulatory arrest time (min)	25 ± 7	24.8 ± 7.6	26.5 ± 5.8	0.385
Cardiopulmonary bypass time (min)	167 (151–191)	167 (150–191)	167 (155–200)	0.705
**Concomitant procedures**				
**Proximal procedures**				
Bentall procedure	43 (54.4)	30 (50.0)	13 (68.4)	0.160
David procedure	3 (3.8)	2 (3.3)	1 (5.3)	0.567
Ascending aortic replacement	23 (29.1)	20 (33.3)	3 (15.8)	0.142
MV repair	3 (3.8)	2 (3.3)	1 (5.3)	0.567
CABG	5 (6.3)	4 (6.7)	1 (5.3)	0.823
**Aortic arch branches**				
Island technique	47 (59.5)	37 (61.7)	10 (52.6)	0.484
Separate	32 (40.5)	23 (38.3)	9 (47.4)	0.484
Extra-anatomic bypass	4 (5.1)	3 (5.0)	1 (5.3)	0.675

*IQR, interquartile range; SD, standard deviation; MV, mitral value; CABG, coronary artery bypass grafting*.

The median CPB and aortic cross-clamp times were 167 (151–191) min and 89 (75–119) min, respectively (Table 2). The mean rectal temperature and circulatory arrest time were 25.3 ± 2.0°C and 25 ± 7 mins, respectively, and the median nasopharyngeal temperature was 23.6 (22.1–24.3)°C.

### Morbidity and Mortality

The early postoperative results are presented in Table 3. Four patients (5.1%) died within 30 days. Two patients died of stroke; 1 patient suffered from heart failure and renal failure postoperatively, and it was difficult to maintain their blood pressure. The patient eventually died; 1 patient suffered from respiratory failure, renal failure and intestinal bleeding, his family members requested transfer to a local hospital, and the patient eventually died.

**Table 3:** Postoperative results.

**Variables**	**Total (** ***n*** **= 79)**	**Subacute (** ***n*** **= 60)**	**Chronic (** ***n*** **= 19)**	***P*** **-value**
Operation death, n (%)	4 (5.1)	3 (5.0)	1 (5.3)	0.675
ICU stay ≤ 3 d, n (%)	66 (83.5)	50 (83.3)	16 (82.4)	0.928
Mechanical ventilation (h), median (IQR)	16.3 (12.0–19.5)	15.5 (11.8–18.9)	17.4 (14.7–31.7)	0.130
Spinal cord injury, n (%)	3 (3.8)	2 (3.3)	1 (5.3)	0.567
Stroke, n (%)	2 (2.5)	1 (1.7)	1 (5.3)	0.426
Acute renal failure, n (%)	4 (5.1)	3 (5.0)	1 (5.3)	0.567
Low cardiac output syndrome, n (%)	1 (1.3)	1 (1.7)	0	0.759
Pulmonary complication, n (%)	2 (2.5)	1 (1.7)	1 (5.3)	0.426
Poor wound healing, n (%)	2 (2.5)	1 (1.7)	1 (5.3)	0.426
Injury to recurrent nerves	0	0	0	–
Limb ischemia	0	0	0	–
**Reoperation**
Reoperation for bleeding, n (%)	5 (6.3)	4 (6.7)	1 (5.3)	0.823
TEVAR, n (%)	2 (2.5)	1 (1.7)	1 (5.3)	0.426
Drainage of pericardial sac, n (%)	2 (2.5)	2 (3.3)	0	0.574

*ICU, intensive care unit; IQR, interquartile range*.

Reoperation for bleeding occurred in 5 patients (6.3%). Two patients (2.5%) required TEVAR because of intimal tears distal to the FET and poor reopening in the distal part of the FET. Two patients (2.5%) required resternotomy because of acute cardiac tamponade. Four patients (5.1%) experienced renal failure and needed continuous renal replacement therapy (CRRT). Spinal cord injury occurred in 3 patients (3.8%), including two cases of paraparesis and one of paraplegia. Poor wound healing occurred in 2 patients (2.5%). There was no recurrent laryngeal nerve injury case. The median time of mechanical ventilation was 16.3 (12.0–19.5) h. Most patients (83.5%) stayed in the intensive care unit within 72 h. The detailed parameters are presented in Table 3.

### Follow-Up

**Figure 3:**
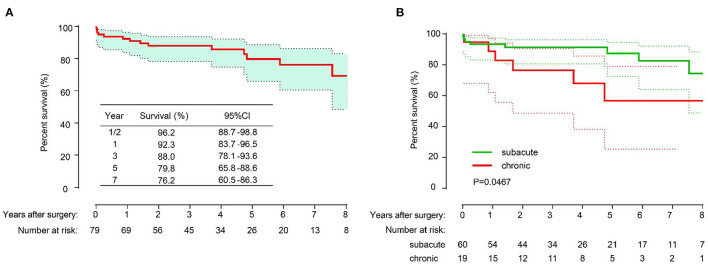
The long-term overall survival of all patients **(A)**; Freedom from distal aortic reoperation of all patients **(B)**.

**Figure 4:**
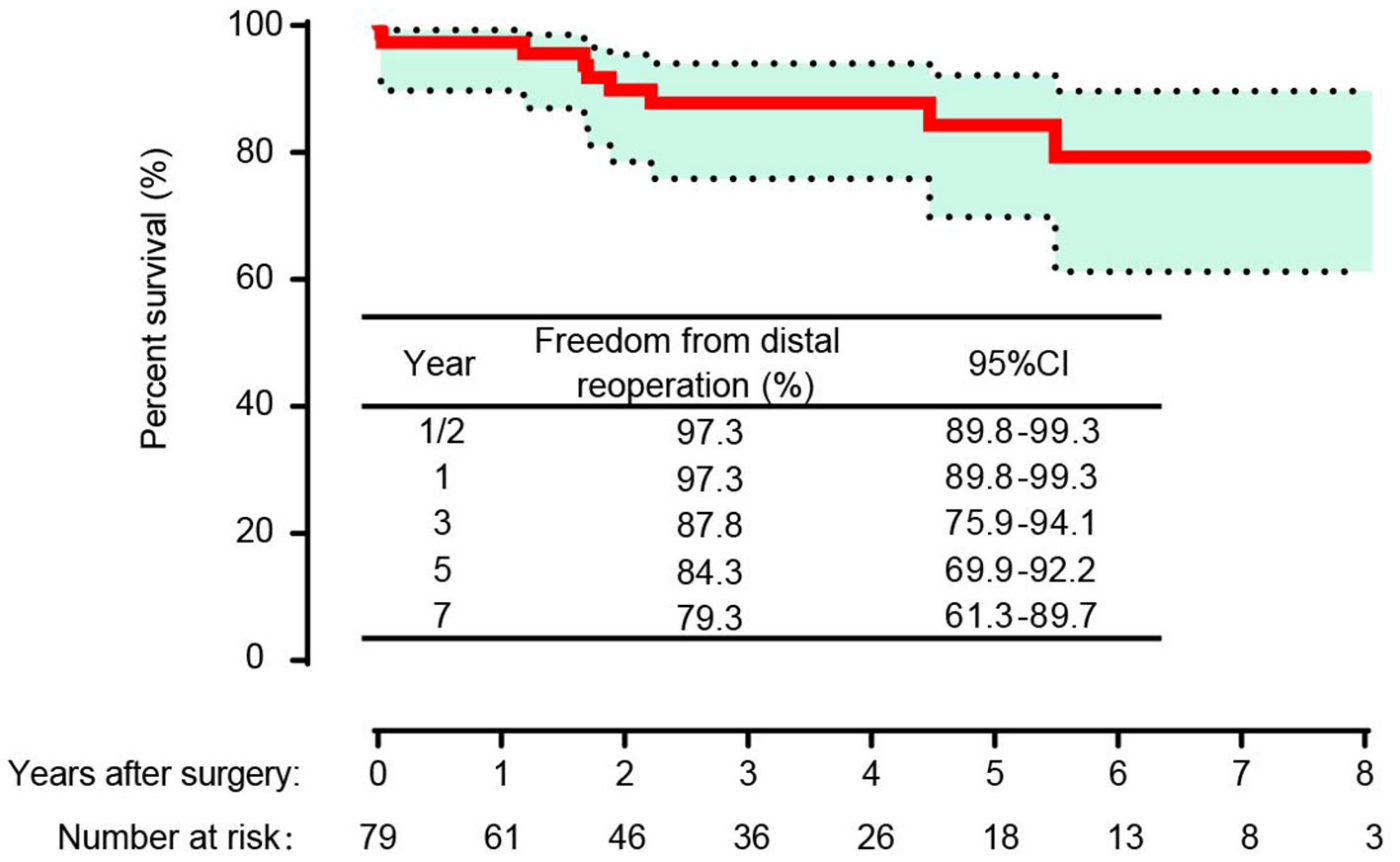
Survival in patients with subacute and chronic type B aortic dissection and compared with the log-rank test.

Among the 75 patients, 66 patients (88.0%) completed the follow-up. The median follow-up time was 53 months [range 3–144 months; 95% confidence interval (CI) 37.88–67.12]. A total of 10 patients died during the follow-up period, with causes that included aortic-related events (*n* = 5), cerebral hemorrhage (*n* = 2) and sudden death (*n* = 3) for unknown reasons. The overall survival rates were 96.2% (95% CI, 88.7–98.8%), 92.3% (95% CI, 83.7–96.5%), 88.0% (95% CI, 78.1–93.6%), 79.8% (95% CI, 65.8–88.6%) and 76.2% (95% CI, 60.5–86.3%) at 1/2, 1, 3, 5, and 7 years, respectively (Figure 3A). The overall survival rate of patients in the subacute group was higher than that in the chronic group (*P* = 0.0467) (Figure 3B). The freedom from distal operation rates were 97.3% (95% CI, 89.8–99.3%), 97.3% (95% CI, 89.8–99.3%), 87.8% (95% CI, 75.9–94.1%), 84.3% (95% CI, 66.9–92.2%) and 79.3% (95% CI, 61.3–89.7%) at 1/2, 1, 3, 5, and 7 years, respectively (Figure 4).

**Figure 5:**
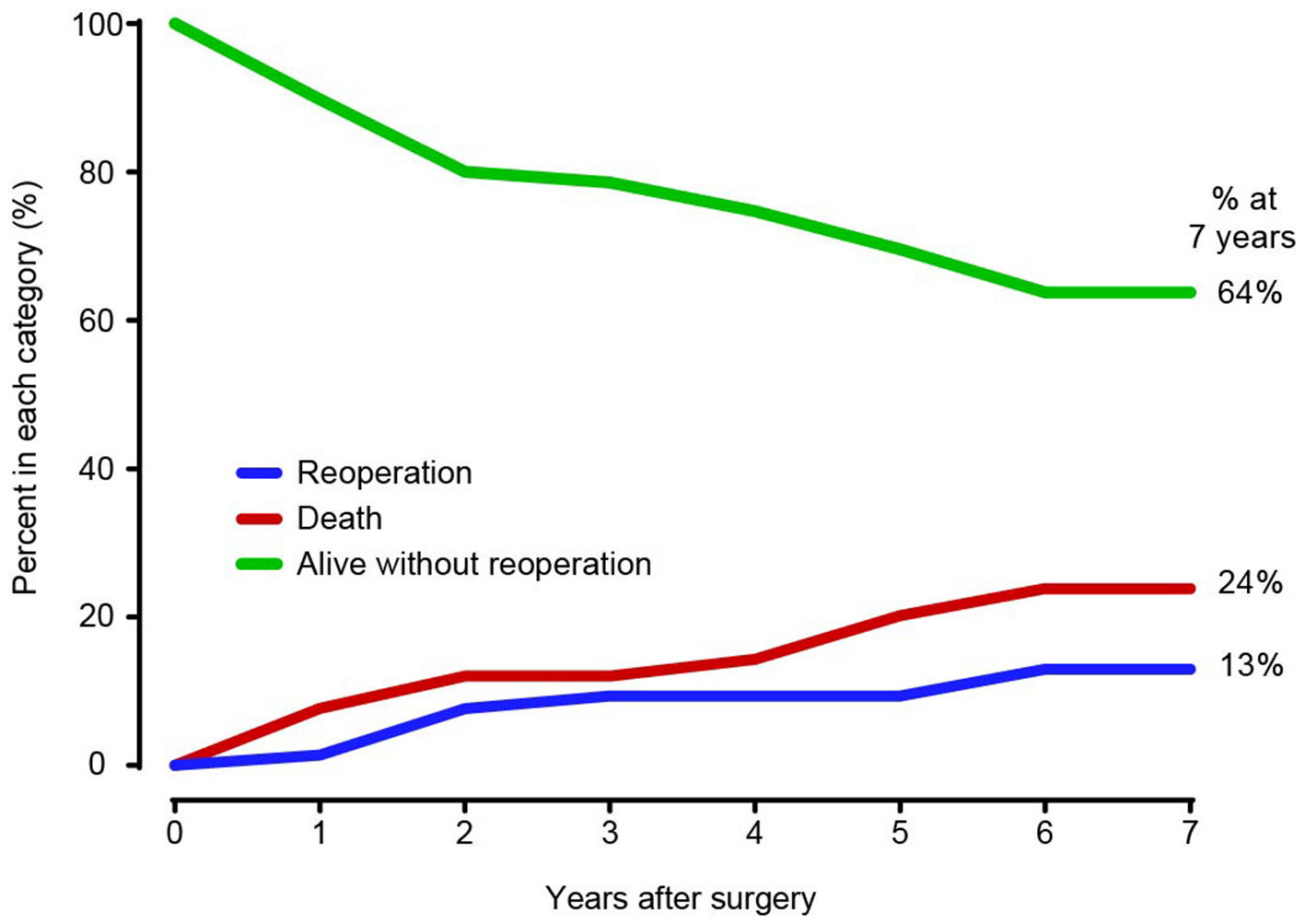
Competing risks of death and distal aortic reintervention.

In the competing risk analysis, the incidence rates of distal aortic reintervention at 7 years were 13 and 24% for late mortality and 64% for survival without distal aortic reoperation (Figure 5). A total of 11 patients underwent distal aortic intervention during the follow-up period. Thoracoabdominal aortic aneurysm repair was performed in 8 patients. TEVAR was performed in two patients for residual dissection and anastomotic leak. One patient underwent EVAR due to an abdominal aortic aneurysm.

## Discussion

The FET procedure is an attractive method for patients with complicated chronic type B or non-A non-B AD who are unfit for TEVAR. The early and long-term outcomes after the FET procedure are favorable.

In current recommendations, TEVAR is considered to be the optimal treatment for complicated type B AD. However, careful evaluation should be performed to determine whether there are enough proximal landing zones and favorable aortic anatomy. Open surgery may be the optimal option for patients with complicated chronic type B AD concomitant with aortic arch aneurysms or proximal aortic lesions. In patients diagnosed with non-A non-B AD with LCCA involvement only, LSCA to LCCA transposition or bypass should be performed, followed by TEVAR (17), and the outcomes are also satisfactory. However, in cases with aortic arch entry or LSCA to LCCA transposition that does not provide a sufficient proximal landing zone, double transposition or total arch rerouting should be considered, but it would also increase the risk of RTAAD (18, 19). According to previous reports, the high-risk factors for RTAAD include ascending aorta >38 mm, bicuspid aortic valve, arch abnormalities and extensive ascending aortic length (5). For such patients, total arch replacement combined with FET implantation was performed in our center. In this study cohort, most patients (78.5%) had concomitant root/ascending aortic aneurysms, and some had aortic arch aneurysms or valvular lesions. Therefore, our center adopted open surgery to perform the FET procedure, and proximal aortic or valvular lesions were treated.

We recommend that the FET procedure be performed if the patients have type B or non-A non-B AD concomitant with the following conditions (20): (i) aortic root or ascending aorta aneurysm; (ii) left common carotid artery dissection involvement or aortic arch aneurysm; (iii) proximal aortic lesion with coronary artery disease or aortic valve disease; (iv) contraindication for TEVAR; and (v) a high risk of RTAAD. The FET procedure for treating type B or non-A non-B AD unfit for TEVAR has some advantages (21). The greatest characteristic was that it combined the advantages of open surgical repair and interventional techniques. First, aortic arch replacement, descending aortic dissection repair and proximal aortic lesion repair can be performed through a median sternotomy in one stage. Second, FET implantation can enlarge the true lumen, promote aortic remodeling and potentially avoid secondary thoracic and abdominal aortic replacement. Third, the FET has extra suture margins at both ends, and anastomosis can be performed for patients who need thoracic and abdominal aortic replacement. Fourth, FET provides a landing zone for secondary TEVAR for those who develop distal stent graft-induced new entry.

The overall in-hospital mortality was 5.1%, which was acceptable for complicated chronic type B or non-A non-B AD, suggesting that the FET procedure is a safe and feasible technique. The overall in-hospital mortality was comparable or superior to those in previous reports (21–24). Takagi et al. (22) reported that the overall in-hospital mortality of chronic type B AD was 3%. Nozdrzykowski et al. (23) reported a single-center experience with 15 patients diagnosed with chronic type B AD who underwent open surgery, and the overall in-hospital mortality was 13.4%. Recently, Weiss et al. (24) reported a retrospective multicenter experience with 57 patients, and the overall in-hospital mortality was 14%. Interestingly, the overall survival rate in the subacute group was superior to that in the chronic group (*P* = 0.047), which contrasted with a previous report (24). Weiss et al. (24) showed that the overall survival rates between acute and chronic AD were not significantly different (*P* = 0.65). Recently, our center also reported experience with chronic type A aortic dissection, and the survival rate showed a distinction between the subacute and chronic groups, although there was no significant difference (*P* = 0.107) (25). The possible explanation may be that chronic B-type dissection itself is often accompanied by dilatation of the descending aorta, which predisposes patients to aortic events. However, this result still needs to be verified objectively, and large-scale studies should be conducted.

Paraplegia remains the most severe complication in aortic surgery because it has serious impacts on the quality of life of patients. In this study, the incidence of spinal cord injury, including paraplegia and paraparesis, was 3.8%, which was similar to the results reported by Weiss et al. (4.0%) (24). Moreover, Preventza et al. (26) found that the incidence of spinal cord injury was 4.7% in a meta-analysis of more than 3,000 patients. Minimizing the circulatory arrest time and FET length above T8 are the key factors in reducing the incidence of spinal cord injury. Furthermore, for high-risk patients, cerebrospinal fluid drainage and neuromonitoring should be performed (27). The incidence of stroke was 2.5%, but all patients with stroke died within 30 days. The results of the meta-analysis indicated that the incidence of stroke was 4.9–6.5% in patients who underwent the FET procedure (28, 29). The presence of bovine aortic arch, preoperative cardiopulmonary resuscitation, aortic valve insufficiency (moderate and severe) and dissection of the common carotid artery were risk factors for postoperative stroke (30, 31). In our study cohort, the incidence of stroke was low compared to those in previous reports (28, 29), which might be due to the small amount of common carotid artery dissection involvement and careful brain protection. Most patients underwent “island technique” arch reconstruction, which preserved autologous vessels and might also have a protective effect on postoperative stroke. However, stroke remains the most devastating complication and warrants further brain protection, including moderate or deep hypothermia, ASCP, cerebral oxygen saturation monitoring and slowing of the rewarming speed (32). Postoperative acute kidney failure is the most common complication following dissection surgery. Minimizing the circulatory arrest time, CPB time and operation time are the key factors to prevent acute kidney failure. For patients with acute renal failure after surgery, early CRRT is recommended, which can partially reverse renal function. The renal resistive index may be helpful for decision-making and improving the prognosis of patients with acute kidney failure (33).

In this series, the overall survival rates and rates of freedom from distal aortic reoperation were 92.3, 79.8, 76.2, and 97.3, 84.3, 79.3% at 1, 5, and 7 years, respectively. There was no cerebral infarction or paraplegia reported. The early and long-term results of this technique for patients with chronic type B or non-A non-B AD were acceptable, which indicates that the FET procedure is a safe and effective approach for such patients. The competing analysis showed that the need for distal aortic reintervention was 13.0% at 7 years, which was comparable with the result reported by Charchyan et al. (20). The authors showed that the cumulative incidence rates of aortic reintervention were 5.6% at 0.5 years and 11.1% at 4 years. Moreover, Weiss et al. (24) showed that 16% of patients with complicated type B aortic dissection required secondary aortic reinterventions at 3 years. In our cohort, it is worth noting that most of the patients who needed reintervention required thoracoabdominal aortic replacement in the follow-up. Moreover, most of the patients (7/8) who required thoracoabdominal aortic replacement had Marfan syndrome, which reminded us of the importance of clinical surveillance and imaging, especially in cases of Marfan syndrome.

## Limitations

There are several limitations to the current study. The present study is a retrospective cohort study, which has inherent selection bias. Moreover, it is a single-center study with a small sample size. Studies with larger groups of patients and multiple center trials should be conducted.

## Conclusion

The FET procedure is a safe and effective approach for treating complicated chronic type B or non-A non-B AD in patients in whom TEVAR is infeasible. This technique combines the advantages of open surgical techniques and endovascular intervention, allowing simultaneous proximal aortic and aortic arch repair and stabilization of the descending aorta. The early and long-term outcomes were acceptable for such patients. The overall survival rate in the subacute group showed superior outcomes compared with the chronic group, but the results should be objectively understood.

## Data Availability Statement

The raw data supporting the conclusions of this article will be made available by the authors, without undue reservation.

## Ethics Statement

The studies involving human participants were reviewed and approved by The Ethics Committees of Beijing Anzhen Hospital, Capital Medical University (2020100X). The patients/participants provided their written informed consent to participate in this study.

## Author Contributions

CL contributed to the conception, data collection, analysis, and drafted the manuscript. RQ, YZ, and SC contributed to the analysis. HL, RG, and YG contributed to the data collection. LS contributed to the conception and writing editing. JZ contributed to the conception, design, acquisition of work, and critically revised the manuscript. All authors approved the submitted version.

## Funding

This work was supported by the Beijing Major Science and Technology Projects from the Beijing Municipal Science and Technology Commission (No. Z191100006619093) and the Natural Science Foundation of China (No. 81970393).

## Conflict of Interest

The authors declare that the research was conducted in the absence of any commercial or financial relationships that could be construed as a potential conflict of interest.

## Publisher's Note

All claims expressed in this article are solely those of the authors and do not necessarily represent those of their affiliated organizations, or those of the publisher, the editors and the reviewers. Any product that may be evaluated in this article, or claim that may be made by its manufacturer, is not guaranteed or endorsed by the publisher.
